# Exploring the cognitive assessment potential of MRI‐based volumetric hippocampal segmentation in Parkinson's disease

**DOI:** 10.1002/brb3.3576

**Published:** 2024-07-05

**Authors:** Merve Tarhan, Başak Atalay, Ayşenur Buz Yaşar, Fatma Betül Özdilek

**Affiliations:** ^1^ Department of Radiology Istanbul Medeniyet University Göztepe Training and Research Hospital Istanbul Turkey; ^2^ Department of Radiology İzzet Baysal State Hospital Bolu Turkey; ^3^ Department of Neurology Istanbul Medeniyet University Göztepe Training and Research Hospital Istanbul Turkey

**Keywords:** hippocampus, magnetic resonance imaging, Parkinson disease

## Abstract

**Purpose:**

To investigate the potential of magnetic resonance imaging (MRI)‐based total and segmental hippocampus volume analysis in the assessment of cognitive status in Parkinson's disease (PD).

**Methods:**

We divided participants into three groups Group A‐Parkinson patients (Pp) with normal cognitive status (*n* = 25), Group B‐Pp with dementia (*n* = 17), and Group C‐healthy controls (*n* = 37). Three‐dimensional T1W Fast Spoiled Gradient Recalled Echo images were used for Volbrain hippocampus subfield segmentation. We used the “Winterburn” protocol, which divides the hippocampus into five segments, Cornu Ammonis (CA),CA2/CA3, CA4/dentate gyrus, stratum radiatum, lacunosum, and moleculare, and subiculum.

**Results:**

A total of 79 participants were included in the study, consisting of 42 individuals with PD (64.2% male) and 37 healthy controls (54.1% male). The mean age of PD was 60.9 ± 10.7 years and the mean age of control group was 59.27 ± 12.3 years. Significant differences were found in total hippocampal volumes between Group A and B (*p* = .047. Statistically significant group differences were found in total, right, and left CA1 volumes (analysis of variance [ANOVA]: *F*(2,76) = 8.098, *p* = .001; *F*(2,76) = 7.628, *p* = .001; *F*(2,76) = 5.084, *p* = .008, respectively), as well as in total subiculum volumes (ANOVA: *F*(2,76) = 4.368, *p* = .016). Post hoc tests showed that total subiculum volume was significantly lower in individuals with normal cognitive status (0.474 ± 0.116 cm^3^) compared to healthy controls (0.578 ± 0.151 cm^3^, *p* = .013).

**Conclusion:**

Volumetric hippocampal MRI can be used to assess the cognitive status of Pp. Longitudinal studies that evaluate Pp who progress from normal cognition to dementia are required to establish a causal relationship.

## Graphical abstract

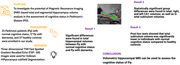

## 1 INTRODUCTION

Parkinson's disease (PD) is characterized by loss of dopaminergic neurons in the substantia nigra and accumulation of Lewy bodies and PD dementia is a result of Lewy body deposition in limbic and neocortical areas, potentially accompanied by neurofibrillary tangles and senile plaques. Dysfunction of non‐dopaminergic neurotransmitter systems, particularly the cholinergic system, is also implicated. Additionally, disruption of frontostriatal circuits linking the basal ganglia with cortical regions contributes to non‐motor symptoms like cognitive impairment, depression, anxiety, apathy, hallucinations, and executive dysfunction (Tanik et al., 2016; Wang et al., 2019; Williams‐Gray et al., 2007). Mild cognitive impairment and dementia usually develop in the later stages in approximately 80% of patients without initial cognitive impairment. PD‐related dementia is the most common cause of dementia after Alzheimer's disease. Dementia is seen in 25% of patients with PD and this increases the risk of being a nursing home patient and death (Camicioli et al., 2003; Foo et al., 2017). Hippocampal atrophy may be seen in patients with PD and may be associated with cognitive impairment (Heim et al., 2017; Meijer et al., 2017). However, hippocampal atrophy is a finding that can be seen in other neurodegenerative diseases and is not specific to PD, such as in Alzheimer's disease (Cabinio et al., 2018; Heim et al., 2017; Meijer et al., 2017; Tsochatzis et al., 2019). Studies on the role of sub‐areas of hippocampal status have been performed, and negative correlations between cognitive scores and right Cornu Ammonis (CA)1, CA2‐3, CA4, and dentate gyrus (DG) and left CA1 subfields were found (Cabinio et al., 2018). As the hippocampus consists of different cell structures and sections, there are studies aiming to reveal the relationship between total hippocampal atrophy as well as segmental atrophy and cognitive disorders seen more specifically in PD (Camicioli et al., 2003; Foo et al., 2017). PD‐associated dementia can be a more predictable condition, and its harmful consequences can be prevented by identifying the presence of specific segmental atrophy, which can provide information about the cognitive prognosis of PD and can be used as a biomarker with volumetric magnetic resonance imaging (MRI) measurements before cognitive disorders occur (Camicioli et al., 2003; Foo et al., 2017; Saad Yousaf & Daniyal, 2012). This study was designed on the hypothesis that the total and segment volumes of the hippocampus may differ in subgroups of patients with PD, according to their cognitive status, and healthy controls.

## 2 MATERIALS AND METHODS

This prospective study was approved by the İstanbul Medeniyet University Clinical Research Ethics Committee (date of 19.02.2020 and project decision number 2019/0510).

### 2.1 Patient and control group selection

Between March 2020 and January 2021, patients with PD (Parkinson patients [Pp]), aged 35–90 years, diagnosed with PD and followed up by the neurology clinic, without any other known neurological disease, were prospectively invited to participate in the study. Patients who were admitted to the neurology clinic with headache, had a normal neurological examination, had no known neurological disease, were referred for cranial MRI, and were also aged between 35 and 90 years were invited to participate as a healthy control group. Depending on their mental capacity, all participants or their relatives provided informed, written consent. Clinical diagnosis and follow‐up of Pp were both performed by a neurologist experienced in movement disorders. Exclusion criteria were as follows: patients in whom volumetric measurements could not be performed; those with known neurological disease or malignancy; and patients with a pacemaker. The cases participating in the study were divided into patients with PD and controls. Pp were further subdivided into those with normal cognitive status and those with dementia.

Patients with PD were grouped into those with normal cognitive status and those with dementia, and then they were evaluated together with the control group within themselves and by comparison with each other. Group composition was as follows: Group 1, patients with PD with normal cognitive status versus healthy controls, Group 2: patients with PD with normal cognitive status versus patients with PD progressing with dementia, and Group 3: patients with PD progressing with dementia versus healthy controls.

### 2.2 Clinical diagnostic criteria for dementia

Patients with cognitive deficits in at least two of four cognitive domains (attention, memory, visuospatial, and executive functions) also patients with cognitive deficiency sufficiently severe to impair daily life were included in Group B‐Pp with dementia. Therefore, our dementia criteria were ≥1.5 SD below the normative data mean on tests in at least two cognitive domains, self‐reported cognitive impairment, detoriation of functional tasks, and complex activities of daily living (Dubois et al., 2007; Emre et al., 2007).

### 2.3 Mini–mental state examination

Mini–mental state examination (MMSE) is a widely used screening test including eleven questions to assess the patient's various cognitive domains, such as orientation, memory, attention, recall, language, and visual‐spatial skills. The maximum score is 30. Cognitive impairment is stratified into three different levels, mild (a score between 18 and 23), moderate (a score between 12 and 17), and severe (a score less than 12) (Litvan et al., 2012). All patients participating in the study, as well as the control group, were assessed using the MMSE.

### 2.4 Neuropsychological testing (NPT)

The neuropsychological battery included measures in the following cognitive domains:

verbal and visual memory (word list recall, story recall, picture recall, and Wechsler Memory Scale‐Revised (WMS‐R) visual reproduction); executive function (Stroop interference, verbal fluency, letter fluency, category fluency, category switching, trails A and B); attention (Digit Span (subtest of the WMS‐R) and visuospatial function (cube copying, clock drawing); and language (Boston Naming Test‐short form). The test battery was examined by the same experienced neuropsychologist, and it took about an hour for each patient (Golden et al., 2002, Lezak et al., 2004, Wechsler, 1955). NPT was only conducted in the patients with PD.

### 2.5 Unified Parkinson's disease rating scale (UPDRS)

Unified Parkinson's disease rating scale (UPDRS) is used to evaluate the severity and progression of PD. Part I of the UPDRS encompasses the assessment of non‐motor dimensions pertaining to daily living experiences. In contrast, Part II focuses specifically on evaluating the motor aspects of daily living experiences. Part III entails a comprehensive motor examination, encompassing the assessment of cardinal motor symptoms. Lastly, Part IV addresses motor complications associated with PD. Lower scores on the UPDRS indicate less impairment and better motor function, whereas higher scores indicate greater impairment and more severe symptoms (Litvan et al., 2012; Movement Disorder Society Task Force on Rating Scales for Parkinson's Disease, 2003).

### 2.6 MRI protocol

MRI scans of all cases participating in the study were performed on the General Electric OptimaTM MR450w 1.5T device (GE Healthcare). In addition to routine cranial MRI protocols, the three‐dimensional T1‐weighted Fast Spoiled Gradient Recalled Echo (3D T1W FSPGR) sequence (TR/TE: 5412/1.94; flip angle 15°; matrix: 192 × 256; FOV, 250 mm; the number of sagittal slices 284; slice thickness 1 mm) was added to the imaging record, adjusted according to the hippocampus sagittal plane and including the whole brain. Axial and coronal multiplanar reformat images were obtained from sagittal 3D T1W FSPGR sequence images.

### 2.7 Hippocampus segmentation and volume measurement

**FIGURE 1:**
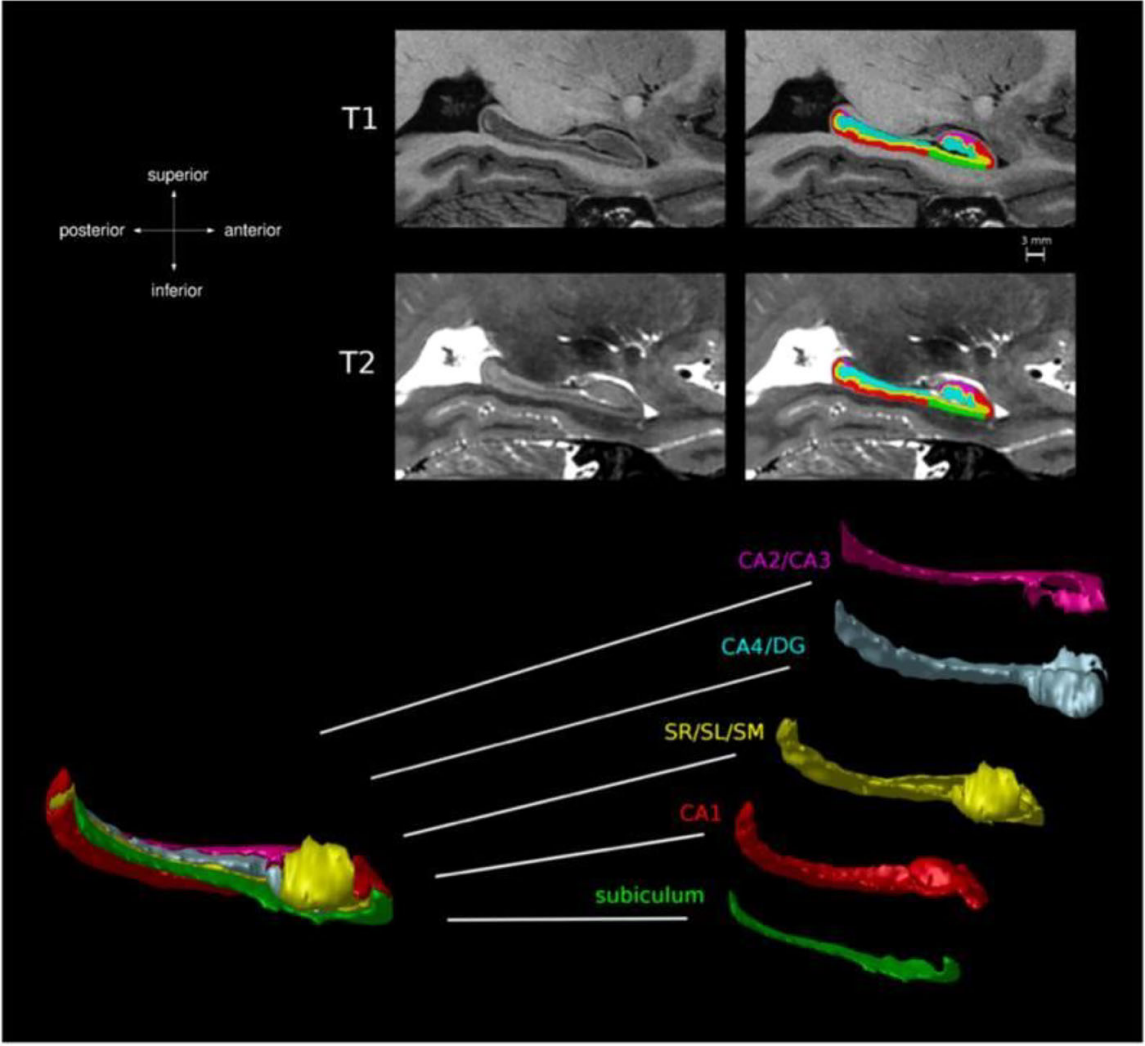
Hippocampus segmentation according to the Winterburn protocol (13): The hippocampus is divided into five segments: Cornu Ammonis (CA)1, CA2/CA3, CA4/dentate gyrus (DG), stratum radiatum, lacunosum, and moleculare (SR/SL/SM) subiculum.

The free online system, Volbrain, version 1.0 (https://volbrain.upv.es), which automates MR brain volumetric measurements, was used to process sagittal, coronal, and axial 3D T1W FSPGR images of the hippocampus and to perform hippocampus segmentation and volumetric measurements. Using Volbrain, hippocampus total and segmental volume measurements were obtained with a method called hippocampus subfield segmentation (HIPS). This method allowed the identification and delineation of specific regions or subfields within the hippocampus. Furthermore, HIPS provided volumetric measurements, which quantify the volume or size of each segmented subfield of the hippocampus, which is hard, due to its complex structure and the lack of high resolution images. HIPS utilizes multi‐atlas label fusion technology, which incorporates a novel multicontrast patch match search process using high‐resolution T1 and T2‐weighted images and a post‐processing step that employs a new neural network‐based error correction technique to minimize systematic segmentation errors (Romero et al., 2017). This approach allowed the researchers to analyze and compare the volume of different subfields within the hippocampus, providing valuable information about the structural characteristics and potential abnormalities in this brain region. We used the “Winterburn” protocol in our study, which divides the hippocampus into five segments, namely, CA,CA2/CA3, CA4/DG, stratum radiatum, lacunosum, and moleculare, and subiculum (Figure 1) (Romero et al., 2017; Winterburn et al., 2013).

**FIGURE 2:**
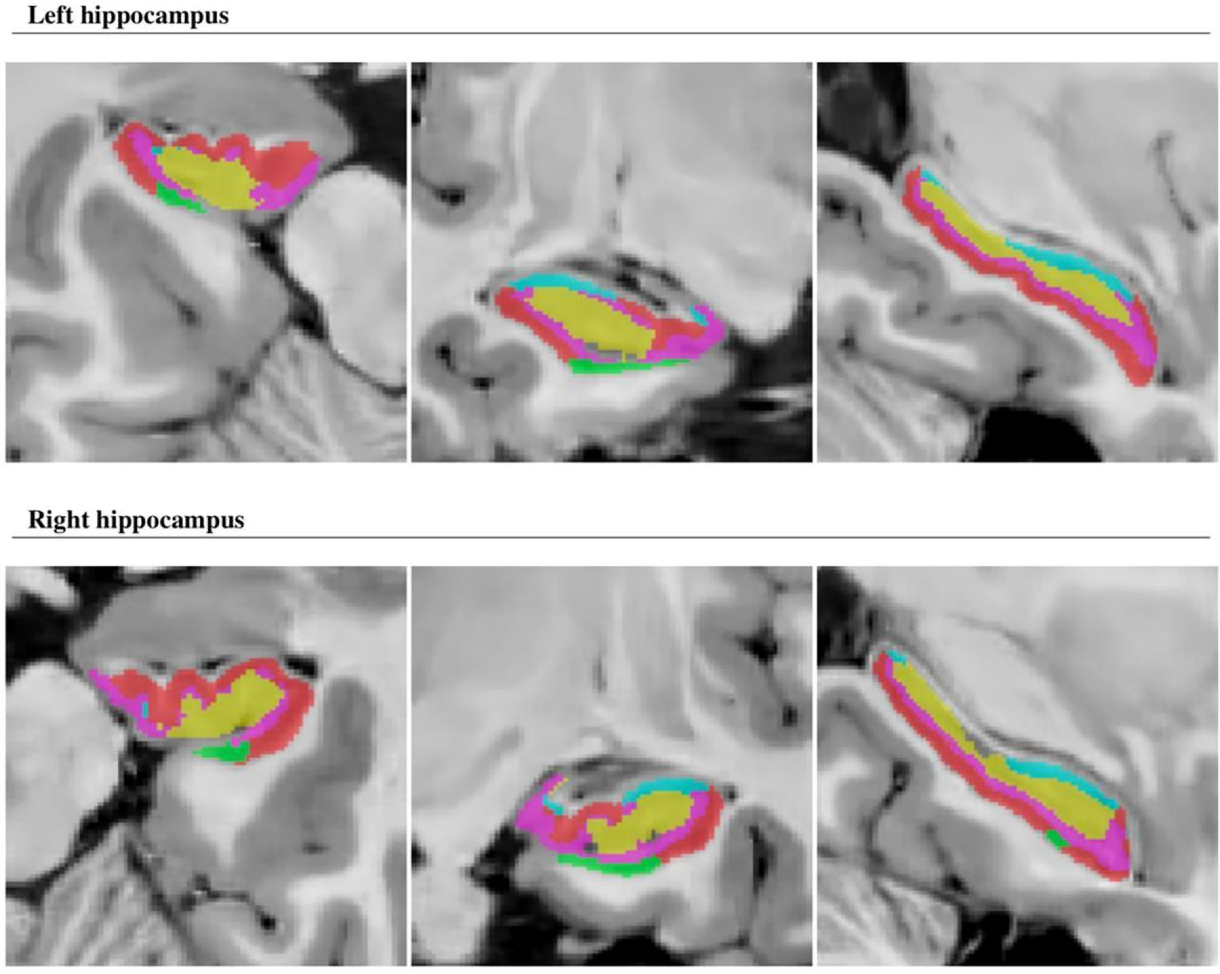
Hippocampal segmentation according to Winterburn protocol for control group. Red: Cornu Ammonis (CA)1, Green: CA2/CA3, Yellow: CA4/dentate gyrus (DG), Blue: stratum radiatum, lacunosum, and moleculare (SR/SL/SM), Pink: Subiculum.

**FIGURE 3:**
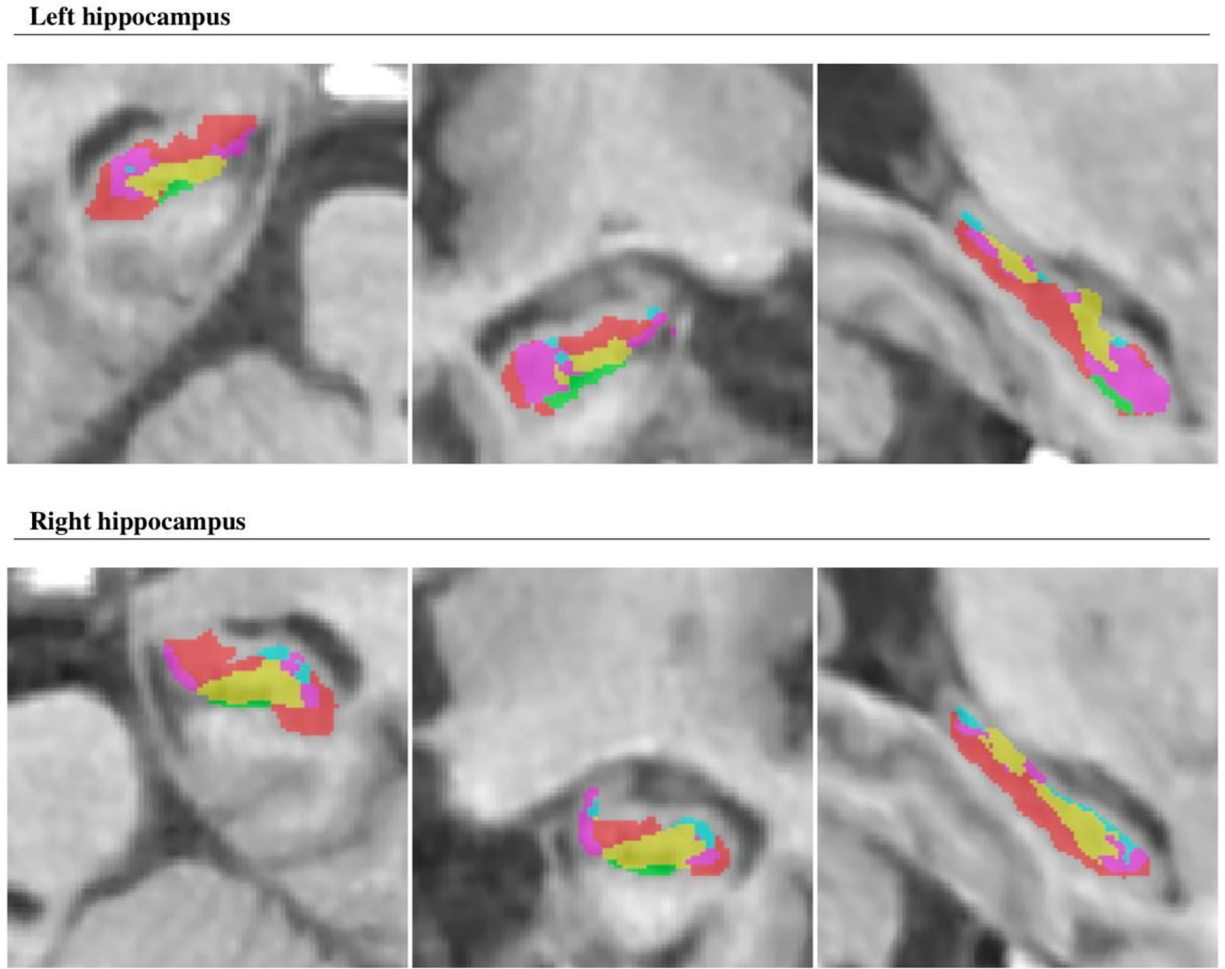
Hippocampal segmentation according to Winterburn protocol for patients with Parkinson's disease. Red: Cornu Ammonis (CA)1, Green: CA2/ CA3, Yellow: CA4/dentate gyrus (DG), Blue: stratum radiatum, lacunosum, and moleculare (SR/SL/SM), Pink: Subiculum.

**FIGURE 4:**
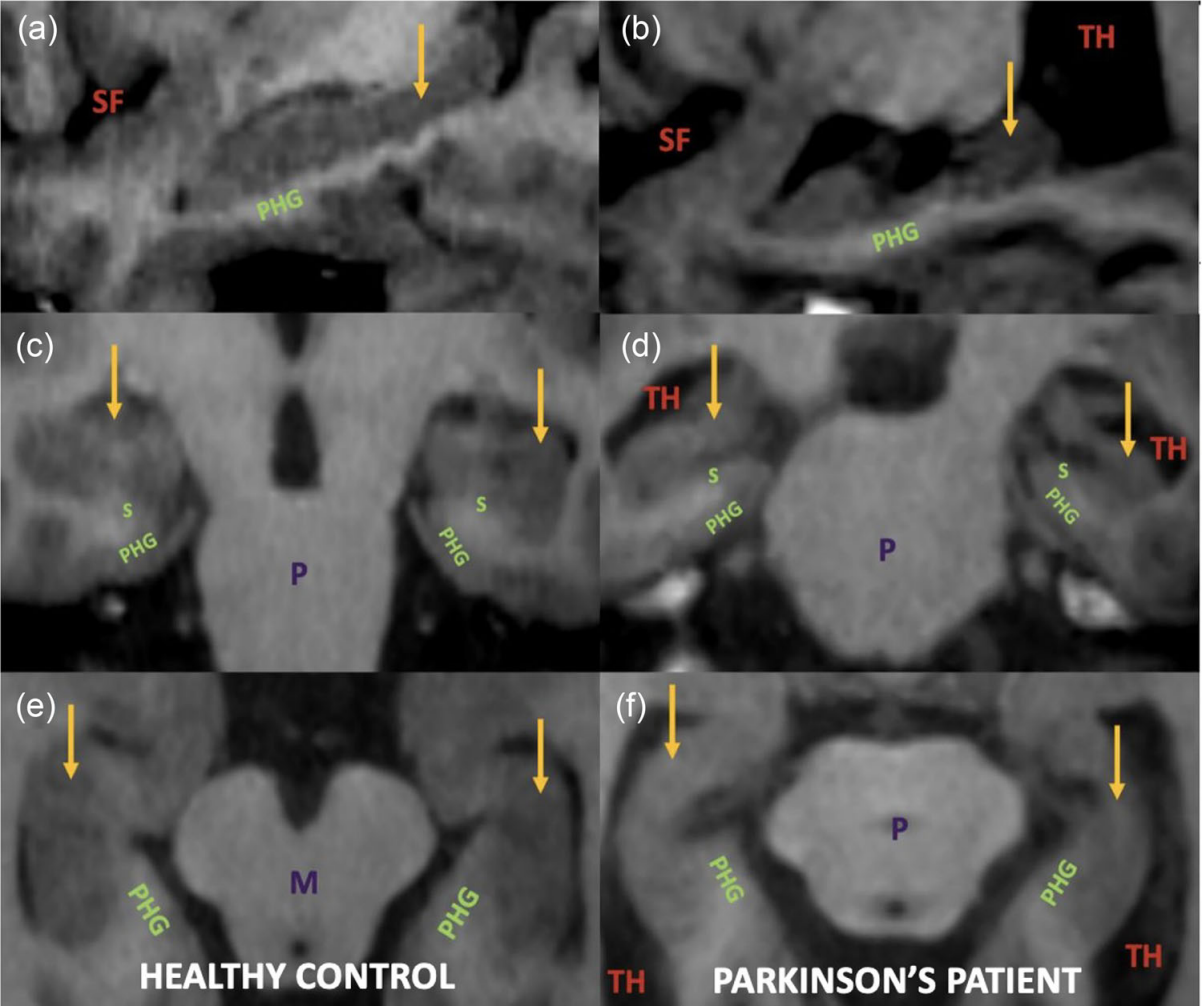
MR image of the hippocampus in healthy control and Parkinson's disease: (a and b) sagittal (c and d) coronal (e and f) axial Fast Spoiled Gradient Recalled Echo (FSPGR) sequence shows the hippocampus (yellow arrow). Compared to the healthy control group (a, c, and e), the hippocampus volume decreases, and atrophy and degeneration occur in Parkinson's disease (PD) (b, d, and f). It is also noteworthy that the subiculum (S) and parahippocampal gyrus (PHG) are also atrophic in PD. In PD, secondary to the hippocampus and cerebral hemisphere atrophy, enlargement of the lateral ventricular temporal horn (TH) and sylvian fissure (SF) is observed. M, mesencephalon; P, pons.

Hippocampal segmentation according to the HIPS‐Winterburn protocol for the healthy control group and Pp is illustrated in Figures 2 and 3. Comparative MR images of the hippocampus in a healthy control and a patient with PD are shown in Figure 4.

### 2.8 Statistical analysis

The normality of data distribution was assessed by Kolmogorov–Smirnov test. However, most data were nonparametric and so the Mann–Whitney *U* test was used to examine demographic differences between Pp with normal cognitive status and Pp with dementia. One‐way analysis of variance (ANOVA) test followed by post hoc test (Tukey‐HSD) was used to compare the relationship between total and segmental volumes of different groups according to the Winterburn protocol. In addition, the effect of demographic and clinical data on total and segmental hippocampus volume in the patient group was evaluated with the chi‐square test for categorical variables and Pearson correlation test for continuous variables. Analysis of Covariance (ANCOVA) is used to adjust for the effects of age. Receiver operating characteristic (ROC) curve analysis was done to test the capacity of the hippocampal atrophy to discriminate between Pp with normal cognitive status and Pp with dementia. Multivariate analysis was performed with the linear regression method with the inclusions of attention and verbal number components of neuropsychological testing (NPT) and education level as covariates in one‐way analysis. The statistical significance level was *p *< .05. Statistical Package for the Social Sciences version 23 program was used for statistical analysis (IBM Inc.).

## 3 RESULTS

### 3.1 Demographic characteristics

A total of 79 patients, 42 with PD (27 men; 64.3%) and 37 controls (20 men; 54.1%), were included. The age range of Parkinson's patients was 37–85, and the mean age was 60.9 ± 10.7, whereas the age range of the control group was between 43 and 81, and the mean age was 59.27 ± 12.3 years. The disease duration varied between 2 and 20 years, with a mean duration of 9.88 ± 4.3 years. In terms of the initial symptomatology, tremor was reported by 57.1% of the patients, whereas the remaining individuals experienced bradykinesia as their first symptom. Five out of the Pp, constituting 11.9% of the total, exhibited a positive familial predisposition toward PD. All the Pp included in the study were right‐handed. The majority of the patients had completed primary school education (76.2%), and only 9.5% of the patients were college or university graduates. On the other hand, 56.7% of the healthy controls had completed primary school education, and only 5.4% of them were college or university graduates. Baseline demographics are summarized in Table 1.

**TABLE 1:** Patient characteristics.

Patient characteristics					
	Parkinson's patients with normal cognitive status (*n* = 25)	Parkinson's patients with dementia (*n* = 17)	All Parkinson's patients (*n* = 42)	Healthy control group (*n* = 37)	*p*‐Value
**Age** (mean in years ± SD)	54.24 ± 1.46	69.94 ± 1.77	60.9 ± 10.7	59.38 ± 12.6	<.001^a^
**Education** (mean in years ± SD)	7.25 ± .75	5.72 ± .57	6.63 ± 1.02	6.49 ± 1.89	.003^a^
**Baseline cognition**					
MMSE score	27.92 ± .26	21.61 ± .34	25.36 ± 2.29	27.3 ± 1.93	<.001^a^
NPT score	49.71 ± 2.5	25.44 ± 2.27	39.92 ± 10.67	N/A	<.001^b^
**Parkinson's disease characteristics**					
UPDRS I	.92 ± .22	1.89 ± .2	1.31 ± .52	N/A	.001^b^
UPDRS II	6.54 ± .86	9.11 ± .77	7.58 ± 1.51	N/A	.047^b^
UPDRS III	9.96 ± 1.6	10.78 ± 1.16	10.29 ± .56	N/A	.196^b^
UPDRS IV	2.58 ± .63	4.22 ± .77	3.24 ± .724	N/A	.036^b^
UPDRS total	20 ± 2.64	25.89 ± 2.31	23.38 ± 3.11	N/A	.044^b^

Abbreviations: MMSE, mini–mental state examination, NPT, neuropsychological testing; UPDRS, unified Parkinson's disease rating scale.

^a^ ANOVA test results of Parkinson patients with normal cognitive status, Parkinson patients with dementia, and healthy controls.

^b^ Independent samples *t* test comparison of Parkinson's patients with normal cognitive status and Parkinson patients with dementia.

### 3.2 Neurocognitive test results

Although MMSE scores of the patients with dementia were lower than Parkinson's patients with normal cognitive status and healthy controls, the patients with dementia scored higher UPDRS scores in all parts of UPDRS. Moreover, NPT scores of the patients with dementia were lower than Pp with normal cognitive status (Table 1).

### 3.3 Hippocampal subfield volumetry

**FIGURE 5:**
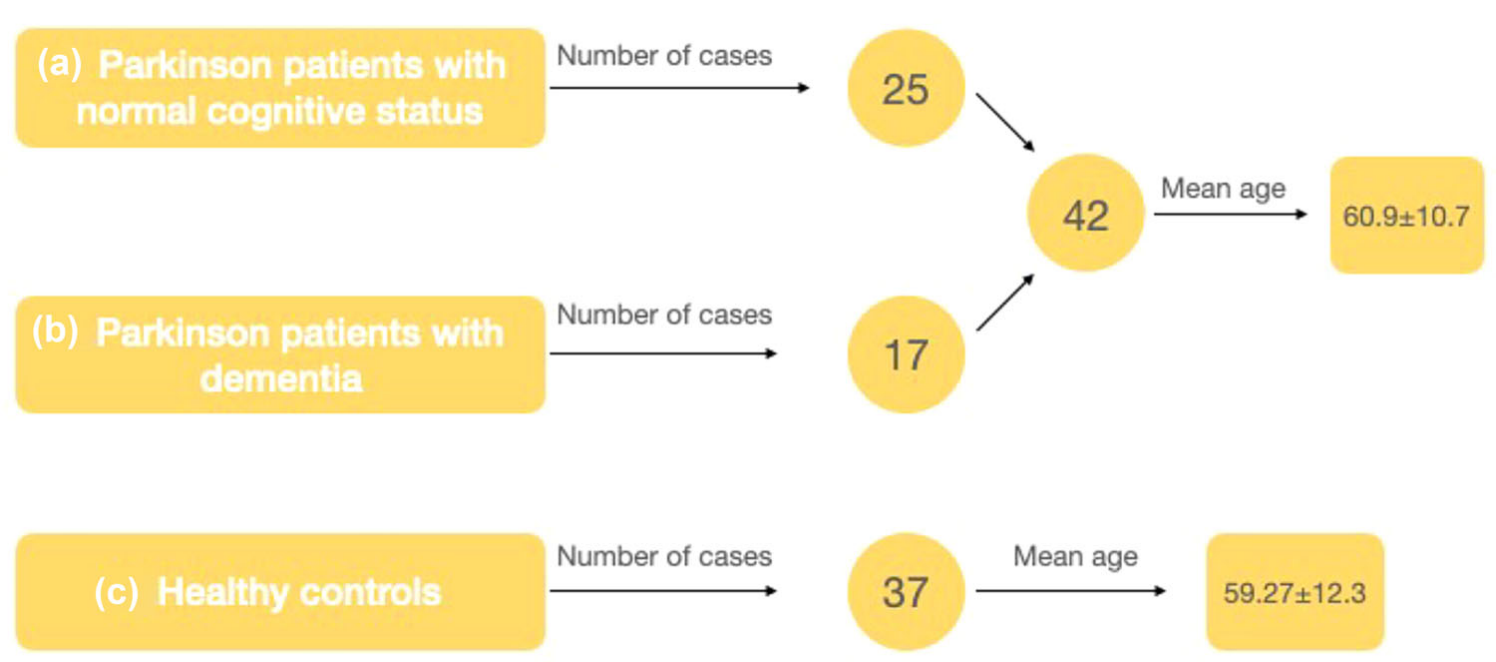
The number of cases participating in different study groups and their average ages.

**FIGURE 6:**
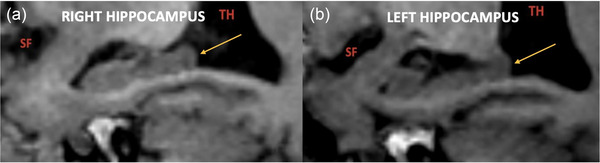
MR image of the right and left hippocampus in a patient with Parkinson's disease (PD): (a) right and (b) left hippocampus (yellow arrow) in the sagittal Fast Spoiled Gradient Recalled Echo (FSPGR) sequence. The left hippocampus volume is less than the right. In both images, enlargement of the lateral ventricular temporal horn (TH) and sylvian fissure (SF) is evident due to hippocampal atrophy.

The number of cases participating in the study groups, their mean age, and hippocampal segmental volumes are summarized in Tables 1, 2, 3 and Figure 5. The mean age of patients with PD and healthy controls was similar, with no significant difference between the mean ages. However, patients with PD progressing with dementia were older than both patients with PD with normal cognitive status and healthy controls (*p*‐value was.001 and.02, respectively). Although not statistically significant, total and segmental volume values in the left hippocampus were generally found to be lower than the right (Figure 6).

**TABLE 2:** One‐way analysis of variance (ANOVA) results.

Volume of hippocampus	Parkinson's patients with normal cognitive status (*n* = 25) cm^3^ mean ± SD	Parkinson's patients with dementia (*n* = 17) cm^3^ mean ± SD	All Parkinson's patients (*n* = 42) cm^3^ mean ± SD	Healthy control group (*n* = 37) cm^3^ mean ± SD	Test of homogeneity of variances		One‐way ANOVA	
					Levene's statistic	*p*‐Value	*F* statistics	*p*‐Value
Total volume	4.233 ± .475	3.696 ± .659	4.016 ± .611	3.942 ± .845	2.087	.131	9.18	.055
Right hippocampus	2.167 ± .282	1.893 ± .333	2.056 ± .329	2.002 ± .497	3.880	.025	2.48	.090
Left hippocampus	2.066 ± .248	1.803 ± .353	1.959 ± .319	1.94 ± .471	2.943	.059	2.341	.103
Total CA1	1.862 ± .263	1.518 ± .343	1.723 ± .34	1.569 ± .34	1.358	.263	8.098	.001
Right CA1	.974 ± .159	.789 ± .197	.899 ± .196	.809 ± .19	1.158	.32	7.628	.001
Left CA1	.849 ± .223	.729 ± .167	.801 ± .209	.76 ± .212	1.821	.169	5.084	.008
Total CA2–CA3	.277 ± .062	.224 ± .091	.256 ± .079	.241 ± .102	3.314	.042	2.119	.127
Right CA2–CA3	.15 ± .029	.120 ± .045	.138 ± .039	.128 ± .061	5.494	.006	2.304	.107
Left CA2–CA3	.127 ± .042	.104 ± .051	.118 ± .047	.113 ± .049	1.099	.338	1.306	.277
Total CA4‐DG	.913 ± .201	.741 ± .252	.843 ± .236	.849 ± .316	2.306	.107	2.033	.138
Right CA4‐DG	.46 ± .116	.371 ± .127	.424 ± .127	.431 ± .181	3.092	.51	1.732	.184
Left CA4‐DG	.453 ± .103	.359 ± .156	.415 ± .134	.418 ± .153	2.252	.112	2.261	.111
Total SR/SL/SM	.686 ± .138	.698 ± .159	.691 ± .145	.706 ± .236	4.57	.013	.074	.929
Right SR/SL/SM	.343 ± .091	.363 ± .089	.351 ± .09	.357 ± .13	2.732	.071	.2	.819
Left SR/SL/SM	.343 ± .091	.335 ± .108	.34 ± .097	.348 ± .131	1.514	.227	.075	.928
Total subiculum	.474 ± .116	.514 ± .14	.383 ± .147	.578 ± .151	.932	.398	4.368	.016
Right subiculum	.241 ± .05	.25 ± .057	.25 ± .07	.278 ± .081	2.042	.137	2.502	.089
Left subiculum	.254 ± .051	.264 ± .092	.166 ± .114	.3 ± .115	1.696	.190	2.013	.141

Abbreviations: CA, Cornu Ammonis; DG, dentate gyrus; SR/SL/SM, stratum radiatum, lacunosum, and moleculare.

**TABLE 3:** Post hoc test (Tukey HSD) results.

Post hoc test (Tukey HSD)				
Volume of hippocampus	Group comparison	Mean difference	*p*‐Value	95% Confidence interval [LL & UL]
Total volume	Parkinson vs. dementia	.537	.047	.005 & 1.069
	Parkinson vs. control	.291	.257	−.147 &.729
	Dementia vs. control	−.246	.464	−.742 &.249
Right hippocampus	Parkinson vs. dementia	.275	.087	−.031 &.58
	Parkinson vs. control	.165	.265	−.086 &.417
	Dementia vs. control	−.109	.631	−.394 &.175
Left hippocampus	Parkinson vs. dementia	.263	.086	−.029 &.555
	Parkinson vs. control	.126	.428	−.115 &.366
	Dementia vs. control	−.137	.454	−.409 &.135
Total CA1	Parkinson vs. dementia	.344	.003	.103 &.585
	Parkinson vs. control	.293	.002	.095 &.492
	Dementia vs. control	−.051	.851	−.276 &.174
Right CA1	Parkinson vs. dementia	.185	.05	.048 &.322
	Parkinson vs. control	.165	.02	.052 &.278
	Dementia vs. control	−.201	.925	−.148 &.108
Left CA1	Parkinson vs. dementia	.159	.018	.022 &.295
	Parkinson vs. control	.128	.022	.016 &.241
	Dementia vs. control	−.031	.831	−.158 &.096
Total CA2–CA3	Parkinson vs. dementia	.03	.133	−.007 &.12
	Parkinson vs. control	.022	.196	−.008 &.092
	Dementia vs. control	−.008	.848	−.079 &.046
Right CA2–CA3	Parkinson vs. dementia	.03	.133	−.007 &.068
	Parkinson vs. control	.022	.196	−.008 &.053
	Dementia vs. control	−.008	.848	−.043 &.027
Left CA2–CA3	Parkinson vs. dementia	.023	.273	−.012 &.059
	Parkinson vs. control	.014	.482	−.015 &.043
	Dementia vs. control	−.009	.798	−.042 &.024
Total CA4‐DG	Parkinson vs. dementia	.172	.116	−.032 &.375
	Parkinson vs. control	.064	.638	−.104 &.231
	Dementia vs. control	−.108	.367	−.298 &.081
Right CA4‐DG	Parkinson vs. dementia	.089	.16	−.026 &.203
	Parkinson vs. control	.029	.742	−.065 &.123
	Dementia vs. control	−.059	.381	−.166 &.047
Left CA4‐DG	Parkinson vs. dementia	.093	.092	−.012 &.199
	Parkinson vs. control	.035	.609	−.052 &.121
	Dementia vs. control	−.059	.327	−.157 &.039
Total SR/SL/SM	Parkinson vs. dementia	−.012	.979	−.158 &.134
	Parkinson vs. control	−.019	.922	−.139 &.101
	Dementia vs. control	−.007	.991	−.143 &.128
Right SR/SL/SM	Parkinson vs. dementia	−.02	.832	−.103 &.063
	Parkinson vs. control	−.014	.869	−.083 &.054
	Dementia vs. control	−.006	.983	−.071 &.083
Left SR/SL/SM	Parkinson vs. dementia	.008	.972	−.078 &.094
	Parkinson vs. control	−.005	.985	−.076 &.066
	Dementia vs. control	−.013	.921	−.093 &.067
Total subiculum	Parkinson vs. dementia	−.04	.623	−.144 &.064
	Parkinson vs. control	−.104	.013	−.189 & −.018
	Dementia vs. control	−.063	.267	−.16 &.033
Right subiculum	Parkinson vs. dementia	−.01	.893	−.06 &.041
	Parkinson vs. control	−.037	.090	−.79 &.005
	Dementia vs. control	−.028	.348	−.075 &.02
Left subiculum	Parkinson vs. dementia	−.01	.935	−.08 &.06
	Parkinson vs. control	−.05	.148	−.105 &.012
	Dementia vs. control	−.04	.401	−.102 &.03

Abbreviations: CA, Cornu Ammonis; DG, dentate gyrus; SR/SL/SM, stratum radiatum, lacunosum, and moleculare.

When total hippocampal volumes were compared between Groups A and B significant differences were found (*p* = .047). There was a statistically significant difference between groups as determined by one‐way for total, right, and left CA1 volumes ANOVA (*F*(2,76) = 8.098, *p* = .001), ANOVA (*F*(2,76) = 7.628, *p *= .001), and ANOVA (*F*(2,76) = 5.084, *p *= .008, respectively). Tukey post hoc tests revealed that the total CA1 subsegment volume was statistically significantly higher in Pp with normal cognitive status (1.862 ± 0.263 cm^3^), compared to Pp with dementia (1.518 ± 0.343 cm^3^, *p *= .003) and healthy controls (1.569 ± 0.34 cm^3^, *p *= .002). There was no statistically significant difference between Pp with dementia and healthy controls (*p *= .851). Right CA1 subsegment volume was statistically significantly higher in Pp with normal cognitive status (0.974 ± 0.159 cm^3^), compared to Pp with dementia (0.789 ± 0.197 cm^3^, *p* = .05) and healthy controls (0.809 ± 0.19 cm^3^, *p *= .02). There was no statistically significant difference between Pp with dementia and healthy controls (*p *= .925). Left CA1 subsegment volume was statistically significantly higher in Pp with normal cognitive status (0.849 ± 0.223 cm^3^), compared to Pp with dementia (0.729 ± 0.167 cm^3^, *p *= .018) and healthy controls (0.76 ± 0.212 cm^3^, *p *= .022). There was no statistically significant difference between Pp with dementia and healthy controls (*p *= .831).

There was a statistically significant difference between groups for total subiculum volumes as determined by one‐way ANOVA (*F*(2,76) = 4.368, *p *= .016). Tukey post hoc tests revealed that total subiculum volume was significantly lower in Pp with normal cognitive status (0.474 ± 0.116 cm^3^) compared to healthy controls (0.578 ± 0.151 cm^3^, *p *= .013). There was no significant difference between Pp with normal cognitive status versus Pp with dementia (0.514 ± 0.14 cm^3^, *p *= .623) and Pp with dementia versus healthy controls (*p *= .267) (summarized in Tables 2 and 3).

According to ANCOVA results, there was a significant main effect of age on hippocampal volume, *F*(2, 76) = 42.076, *p *< .001, and partial *η* ^2^ = .356. For hippocampal volume, estimated mean was 3.981 and standard error was.066 (lower bound 3.851 and upper bound 4.112, 95% confidence interval).

### 3.4 The ability of hippocampal atrophy in discrimination of dementia in PD

**FIGURE 7:**
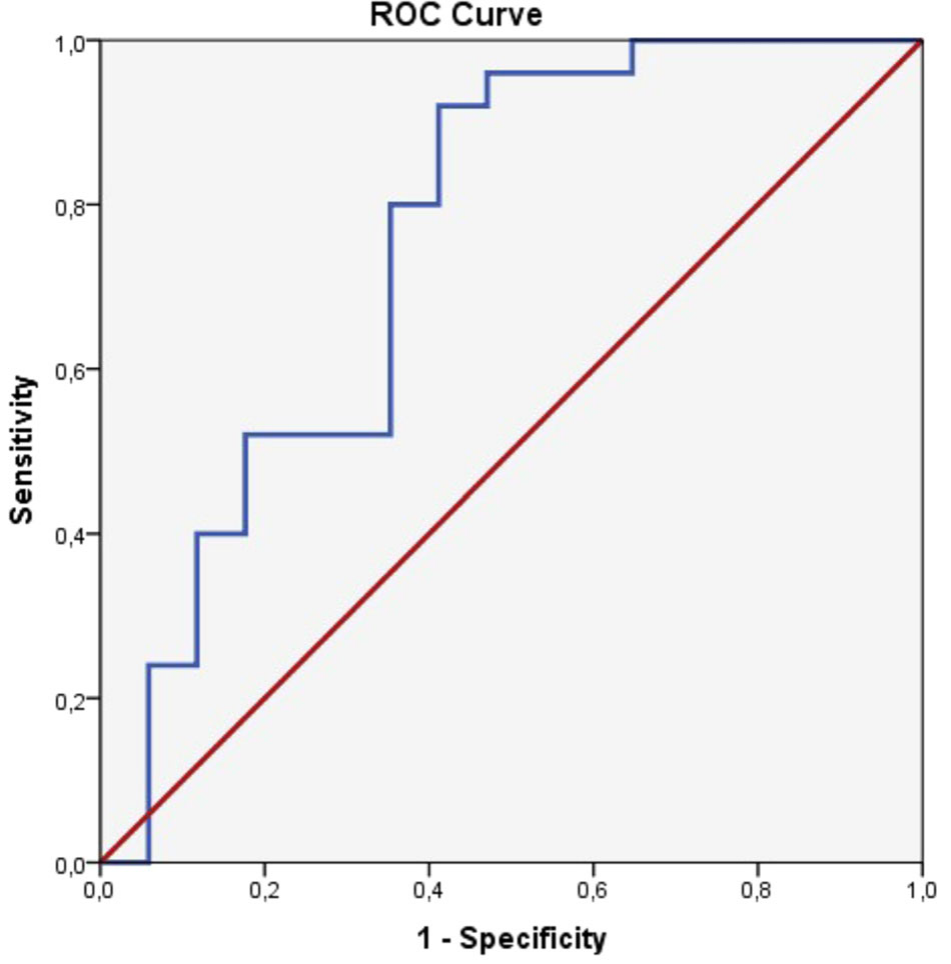
Receiver operating characteristic (ROC) curve analysis of the ability of hippocampal atrophy to predict dementia in Parkinson's disease (PD) (area under the curve [AUC]:.753, cut‐off value 3.86 cm^3^ with a sensitivity of 0.80 and specificity of 0.647).

ROC curve analysis was conducted to evaluate the capacity of hippocampal atrophy to differentiate dementia in PD. ROC curve analysis revealed that area under the curve was.753, *p*‐value was.006. At the optimal cut‐off point (3.86 cm^3^), the sensitivity was 0.80 and the specificity was 0.647 in distinguishing between Pp with normal cognitive status and Pp with dementia (Figure 7). These findings suggest the potential utility of hippocampal atrophy as a predictive tool for dementia.

### 3.5 Association of hippocampal volume and neurocognitive test results and education level

We assessed the relationship between total hippocampus volume, attention and verbal number components of NPT, and education level in patients with PD with a linear regression model. The analysis revealed that attention and verbal number components of NPT had a significant influence on hippocampus volume (*p *< .05).

## 4 DISCUSSION

The hippocampus plays a pivotal role in memory consolidation and various cognitive functions, and its structural integrity is closely linked to cognitive performance (Fortin et al., 2002). Therefore, by examining hippocampal volume in PD patients, we aimed to elucidate potential structural changes associated with cognitive decline, providing insights into the underlying neurobiological mechanisms of cognitive impairment in PD. This assessment not only aids in early detection and monitoring of cognitive dysfunction but also holds promise as a potential biomarker for disease progression and treatment response in PD. Thus the aim was to compare total and segmental hippocampus volumes of patients with PD and healthy controls using MRI. When the hippocampus volumes of Parkinson's patients with normal cognitive status and the control group were compared, the percentage of total subiculum volumes of Pp was found to be significantly lower than the control group. When comparing hippocampus volumes of Pp with normal cognitive status and Pp with dementia, total hippocampus volume and percentage of the total hippocampal volume were found to be significantly lower in the Pp with dementia. PD stems from the unique pattern of onset and progression observed in this neurological disorder. PD typically manifests with unilateral symptoms, affecting one side of the body more severely than the other in the early stages of the disease (Modestino et al., 2016).

Studies have reported that the CA1 region is affected in PD (Foo et al., 2017; İzci & Erbaş, 2015; Lacaille & Schwartzkroin, 1988). There was a statistically significant difference between the groups in terms of CA1 percentiles in our study as well. Aging is associated with a decrease in total hippocampal volume; specific subregions like the CA can also be affected, potentially contributing the major influence on our results. Moreover, subiculum volumes were found to be significantly lower in patients with PD compared to the control group. The subiculum provides the connection between the CA1 segment of the hippocampus and the entorhinal cortex (O'Mara, 2005). We think that the atrophy observed in the subiculum in PD may lead to cognitive disorders due to the effects or connections of these regions on CA1.

The relationship between total hippocampus volume, attention, and verbal number components of NPT in patients with PD who participated in the study was found to be statistically significant. In cases with low total hippocampus volume, impairment in attention, and verbal number functions were observed. In our study, although not statistically significant, left total and segmental hippocampus volumes were generally found to be lower than the right. This situation can be associated with the fact that verbal memory impairment is more prominent in patients with PD, as reported by Bruck (2004). In the study of Yıldız et al. (2013), patients with mild cognitive impairment reduction in right and left hippocampus volumes have been associated with deterioration in word fluency and cognitive status.

Studies have evaluated hippocampal volumes in patients with PD with regard to cognitive function. Specific atrophy of the right CA4, left parasubiculum, DG, and left hippocampal‐amygdala transition region has been identified as potential markers in the progression of PD to mild cognitive impairment (Foo et al., 2017). In discussing cognition, it is imperative to consider factors such as hippocampal volume and white matter lesions as potential confounding variables, which may significantly influence cognitive outcomes and should be carefully accounted for in future research endeavors (Janssen et al., 2007). Another study focused on prediction of dementia progression in idiopathic PD showed that patients who developed dementia exhibited smaller initial global hippocampal volume; also, CA1, subiculum, and presubiculum volumes were lower. They suggest early decrease of CA1, subiculum, and presubiculum volumes as a biomarker to predict dementia conversion (Low et al., 2019).

Camicioli et al. compared hippocampal volumes among different groups: PD patients without cognitive impairment, PD patients with mild cognitive impairment or dementia, Alzheimer's patients, and a control group. The study suggested that progressive hippocampal volume loss may occur in dementia cases and that volumetric MRI examination in PD could serve as an early marker for dementia development (Camicioli et al., 2003).

Uribe et al. studied the hippocampal volumes of the patients with PD in a normal cognitive state, patients with PD with mild cognitive impairment, and the healthy control group at two different time intervals. While a time effect was observed in the right total hippocampus volume in both Parkinson's groups, no change was found in the control group. A time effect was observed in the left hippocampal tail and right parasubiculum volumes, even in patients with PD with normal cognitive status (Uribe et al., 2018). Beyer et al. reported that patients with mild cognitive impairment had decreased volume and correlated attention scores in CA1 at baseline compared to patients with PD with normal cognitive status. These authors proposed that subiculum and CA1 atrophy may be related to the impairments in memory function tests reported in all patients with PD (Beyer et al., 2012).

A recent study involving a 6‐week training program with visually stimulating computer games and physical exercise in PD patients and a control group showed increased volumes in specific areas of the hippocampus in both groups (left CA1, CA4/DG, and subiculum) after the training, highlighting the plasticity of the hippocampus (Schaeffer et al., 2021). Hippocampal volumes can be enhanced with such training programs, regardless of cognitive status, through the ability of neuroplasticity. Hence, we recommend a fully automated volumetric segmentation analysis program for hippocampal segmentation, namely, HIPS, which we utilized in this current study, that could be used in Pp to select candidates for such training programs. In a longitudinal study, conducted by Xu et al. (2020), it is found that PD patients with cognitive decline had significantly smaller bilateral CA4, molecular layer, and granular cell layer of the DG subfields, as well as a smaller left CA2/3 and right presubiculum subfields, compared to PD patients without cognitive decline. In a systematic study in which they examined MRI studies on episodic memory and hippocampus correlations in PD, episodic memory and cognitive impairment in PD were most frequently associated with CA1, CA3/4, and subiculum in hippocampal segment studies (Pourzinal et al., 2021).

Studies have been published about the relationship between cognitive status and hippocampus volume and their follow‐up. In our study, subiculum volumes were found to be significantly lower in the Parkinson group compared to the control group. According to our findings, we suggest that subiculum volumes may have an impact on memory by affecting the connection paths of the hippocampus with other segments or direct segments. We recommend using a volumetric assessment of the hippocampus to assess the development of cognitive impairment or dementia in people diagnosed with PD and to support clinical testing; however, there is no consensus on the total‐segmental volume of the hippocampus that can be used as a biomarker for patients with PD who will develop cognitive impairment or dementia. With the development of MRI technologies and segmentation programs, more accurate measurements and more robust results will become available. Multicenter studies and meta‐analyses with large participation are required to resolve this situation. There are a number of study limitations that should be noted. All patients with cognitive impairment in our study also had progressive dementia. Due to the absence of patients with PD with mild cognitive impairment in the study group, the transition from normal cognition to dementia could not be evaluated. In patients with a normal cognitive status, mild cognitive impairment or dementia may develop during follow‐up. In order to predict or evaluate this situation, the relationship between the changes observed in initial volumes and cognitive function should be evaluated during sufficient follow‐up. There are two different studies conducted using the Volbrain program for volumetric measurement of hippocampus on healthy persons or patients with major cognitive disorders; however, to the best of our knowledge, our study is the first to evaluate hippocampal volume using the Volbrain program in PD (Özdemir et al., 2019; Yücel et al., 2022). Volbrain offers accurate, efficient, and accessible automated analysis of brain MRI data, with multi‐modal capabilities, normative database comparisons, and broad applications in research and clinical settings. Our study highlights the differences in hippocampal volume between Pp with normal cognitive status and those with dementia, as well as the association between neurocognitive test results and hippocampal volume in Pp. These findings provide valuable insights into the neurobiological mechanisms underlying cognitive impairment in PD.

## 5 CONCLUSION

This study contributes to the understanding of the relationship between hippocampal volume, neurocognitive function, and PD. Volumetric hippocampal MRI may be useful to assess segmental atrophy as a biomarker for the development of cognitive impairment and/or dementia in patients with PD over time. The subiculum volumes were significantly different between the PD and control groups, independent of the cognitive status, and quantitative values can be obtained to support the diagnosis of PD without obvious radiological findings. The difference in hippocampus volumes in PD without cognitive impairment compared to the control groups may be attributed to the neurodegenerative background. As a result, larger studies including follow‐up volumetric values are needed to predict the development of cognitive impairment and/or dementia in patients with PD in the future.

### 5.1 Main points

- Progressive hippocampal volume loss may occur in cases with dementia and that volumetric MRI examination in PD may be an early marker for the development of dementia.
- Segmental atrophy of the hippocampus may be used as a biomarker to predict the development of cognitive impairment and dementia in PD patients.
- The subiculum found to be significantly lower in patients with PD compared to control groups may lead to cognitive disorders due to its effects or connections on the CA1 region.

## AUTHOR CONTRIBUTIONS

**Merve Tarhan**: Writing—original draft; investigation; conceptualization; methodology; data curation. **Başak Atalay**: Writing—original draft; conceptualization; methodology; supervision. **Ayşenur Buz Yaşar**: Writing—review & editing; writing—original draft; software; formal analysis. **Fatma Betül Özdilek**: Conceptualization; supervision; formal analysis; data curation.

## CONFLICT OF INTEREST STATEMENT

The authors declare that they have no conflicts of interest. All authors have actively participated in this manuscript and approved the final article.

## FUNDING INFORMATION

The researchers covered the expenses related to this study by themselves.

### PEER REVIEW

The peer review history for this article is available at https://publons.com/publon/10.1002/brb3.3576.

## IRB STATEMENT

This study was performed in accordance with the Declaration of Helsinki, and the institutional committee has approved the prospective design of the study. Written informed consent was provided from all participants.

## Data Availability

The data that support the findings of this study is available on request.
